# An ST elevation myocardial infarction with multisystemic embolization: a shocking and striking first presentation of antiphospholipid syndrome: a case report

**DOI:** 10.1093/ehjcr/ytaf296

**Published:** 2025-06-24

**Authors:** Meryem Haboub, Ilyas Atlas, Abdenasser Drighil, Rachida Habbal

**Affiliations:** Cardiology Department, University Hospital Ibn Rochd, 8, street Lahcen El Arjoun, 20100 Casablanca, Morocco; Cardiology Department, University Hospital Ibn Rochd, 8, street Lahcen El Arjoun, 20100 Casablanca, Morocco; Cardiology Department, University Hospital Ibn Rochd, 8, street Lahcen El Arjoun, 20100 Casablanca, Morocco; Cardiology Department, University Hospital Ibn Rochd, 8, street Lahcen El Arjoun, 20100 Casablanca, Morocco

**Keywords:** Anti-phospholipid syndrome, ST-segment myocardial infarction, Left ventricle thrombus, Arterial embolization, Case report

## Abstract

**Introduction:**

Anti-phospholipid syndrome is characterized by venous and/or arterial thrombosis in the presence of anti-phospholipid antibodies. We report a rare and dramatic manifestation of the syndrome: thrombotic coronary occlusion leading to myocardial infarction, resulting in multiple intra-LV thrombi responsible for multisystemic embolization.

**Case presentation:**

We report the case of a 38-year-old Caucasian woman, who presented to the emergency department with acute chest pain. On initial clinical examination, the patient was tachycardiac at 123 b.p.m. with a correct blood pressure of 127/69 mmHg. The electrocardiogram showed sinus tachycardia at 125 b.p.m. with QS waves in the anteroseptal with persistent ST-segment elevation in the same territory. Transthoracic echocardiography revealed left ventricle ejection fraction of 35% with several intra-left ventricular (LV) thrombi. Troponin Ic was elevated at 5668 ng/L. The diagnosis of myocardial infarction was suspected and the patient was treated as such. Eight hours after admission, the patient presented with an embolization to both common femoral arteries which was treated by Fogarty embolectomy. The patient underwent coronary angiography, which revealed thrombotic occlusion of the proximal left anterior descending artery. A cerebro-thoraco-abdomino-pelvic computed tomography scan found a right renal infarct and a splenic infarct. Lab tests revealed positive anti-cardiolipin antibodies. Anti-phospholipid syndrome was confirmed and the patient was treated using aspirin and vitamin K antagonists. The evolution was marked by complete resolution of LV thrombi and the patient is actually asymptomatic apart from a slight exertional dyspnoea.

**Conclusion:**

Anti-phospholipid syndrome is an autoimmune disorder whose complications can be life-threatening and/or functionally disabling. Arterial thrombosis can cause dramatic complications.

Learning points

- Arterial manifestations of anti-phospholipid syndrome (APS) are less frequent than venous manifestations but are severe.
- An ST-segment elevation myocardial infarction due to thrombotic occlusion of an epicardial coronary artery is possible in the context of APS and the treatment consists of vitamin K antagonists and low-dose aspirin.

## Introduction

Anti-phospholipid syndrome (APS) is an acquired autoimmune thrombophilia characterized by venous and/or arterial thromboses in the presence of anti-phospholipid antibodies (APL).^1^

Arterial manifestations are less frequent than venous thrombosis in APS, but are generally more severe and potentially life threatening.^2^ In the Euro-Phospholipid project registry, stroke and myocardial infarction were the most frequent causes of death from APS (22.5% of cases).^3^

We report a rare and dramatic manifestation of APS: thrombotic coronary occlusion leading to myocardial infarction, resulting in multiple intra-LV thrombi responsible for multisystemic embolization.

## Summary figure

**Table:** 

Time	Event
The day of presentation	Acute chest pain. The electrocardiogram shows ischaemic signs. Transthoracic echocardiogram revealed reduced left ventricle ejection fraction with multiple left ventricle thrombi.
The same day, 8 h after hospital admission	The patient presented with sudden pain in both lower limbs with absent distal arterial pulses in both lower limbs, which were white and cold. Arterial echo-Doppler shows demodulated flow femoral arteries, with no flow in the popliteal, anterior and posterior right and left tibial arteries. The patient was rapidly transferred to the operating room for Fogarty embolectomy. Arterial echo-Doppler control confirms the permeabilization of the arterial tree.
24 h after hospital admission	The patient underwent coronary artery angiography, revealing a thrombotic occlusion of the proximal left anterior descending artery.
Four days after hospital admission	Computed tomography scan was performed as part of the search for subclinical systemic embolisms, finding a right renal infarct and a splenic infarct and confirming the multisystemic embolization.
Six days after hospital admission	The initial work-up requested to look for coagulopathy or thrombophilia revealed positive anti-cardiolipin antibodies (IgG and IgM). The patient was put on vitamin K antagonists with INR monitoring with low-dose aspirin in addition to maintaining the rest of heart failure treatment.
Three weeks later	Transthoracic echocardiogram control revealed complete resolution of LV thrombi.

## Case presentation

We report the case of a 38-year-old Caucasian woman, with no specific pathological history or cardiovascular risk factors, who presented to the emergency department with acute chest pain.

On initial clinical examination, the patient was conscious 15/15 on Glasgow Coma Scale, apyretic, tachycardiac at 125 b.p.m. with a blood pressure within normal range of 127/69 mmHg.

The electrocardiogram showed sinus tachycardia at 125 b.p.m. with QS waves in the anteroseptal with persistent ST-segment elevation in the same territory, in addition to negative T waves in the apical-lateral region.

**Figure 1:**
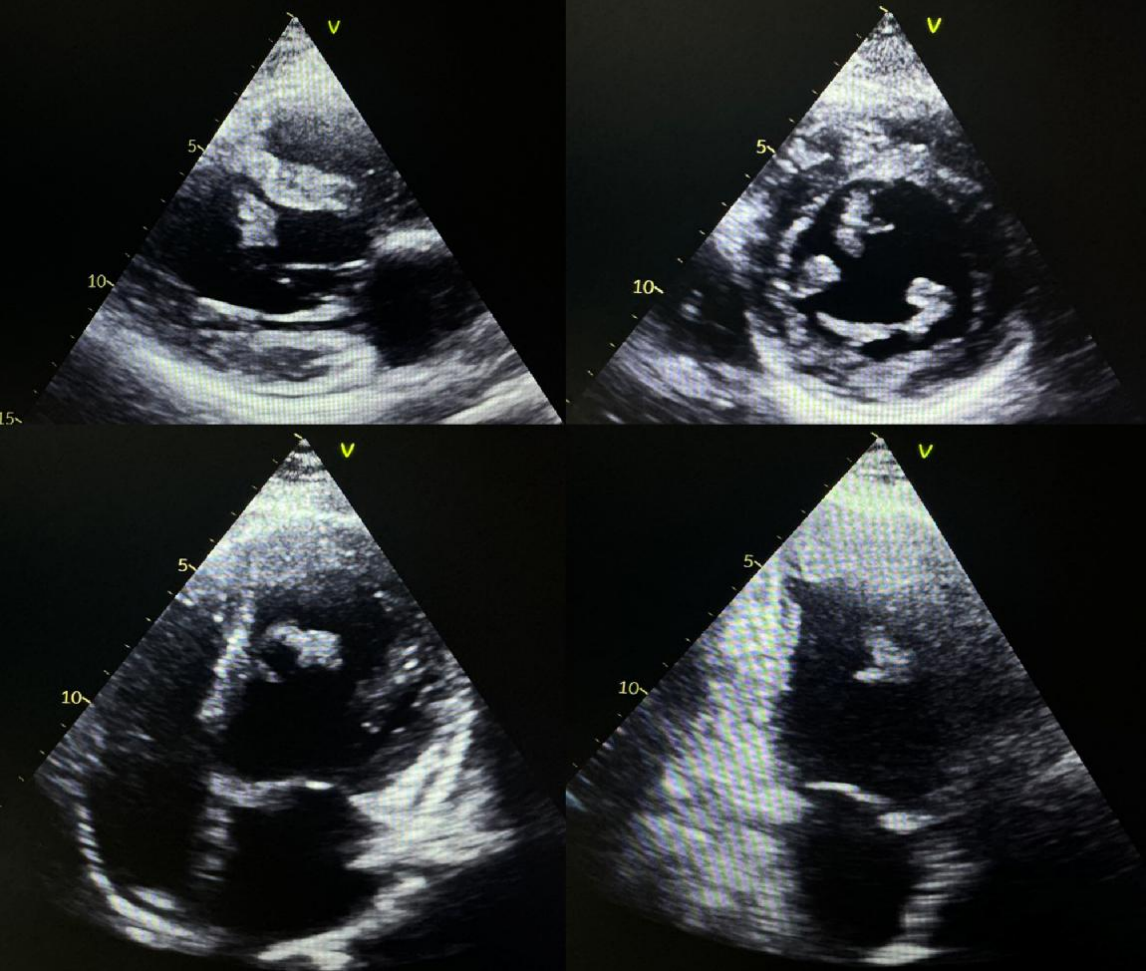
Transthoracic echocardiography images showing intra-LV thrombi.

Transthoracic echocardiography (*Figure 1*) performed on admission revealed akinesia of the LV apex, inferoseptal and anteroseptal walls, with hypokinesia of the anterior and anterolateral walls and a reduced left ventricle ejection fraction (LVEF) of 35%. There were also several intra-left ventrcular (LV) thrombi, some of which were pedunculated and mobile, and a thrombus lining the apex measuring 30 mm in long axis.

The biological work-up revealed that the platelets were 188 000/mm^3^ (normal range: 150 000–450 000/mm^3^), the white blood cells were 15 430/mm^3^ (normal range: 4000–11 000/mm^3^), the haemoglobin level was 13.5 g/dL (normal range: 12–16 g/dL), and the prothrombin level was 81% (normal range: 70–100%). Lipid, thyroid, liver, and renal profiles were all within normal ranges. At 5668 ng/L, troponin Ic was higher than the laboratory normal value, which is <19 ng/L.

Given this situation, the diagnosis of myocardial infarction was suspected, and the patient was treated as such with: Aspirin 100 mg/day, Clopidogrel 75 mg/day, Lansoprazole 30 mg/day, and Bisoprolol 5 mg/day. In addition, we added Ramipril 2.5 mg/day and Spironolactone 25 mg/day as heart failure treatment, in addition to curative dose of unfractionated heparin with strict monitoring of Activated Cephalin Time.

**Figure 2:**
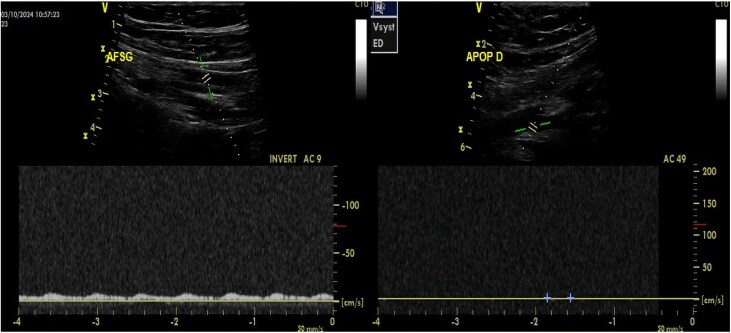
Arterial echo-Doppler images of both lower limbs showing pathological flows.

Eight hours after admission, the patient presented with sudden pain in both lower limbs. On clinical examination, distal arterial pulses were absent in both lower limbs, which were white and cold. An arterial echo-Doppler (*Figure 2*) showed demodulated flow in the common, superficial and deep right and left femoral arteries, with no flow in the popliteal, anterior and posterior right and left tibial arteries.

**Figure 3:**
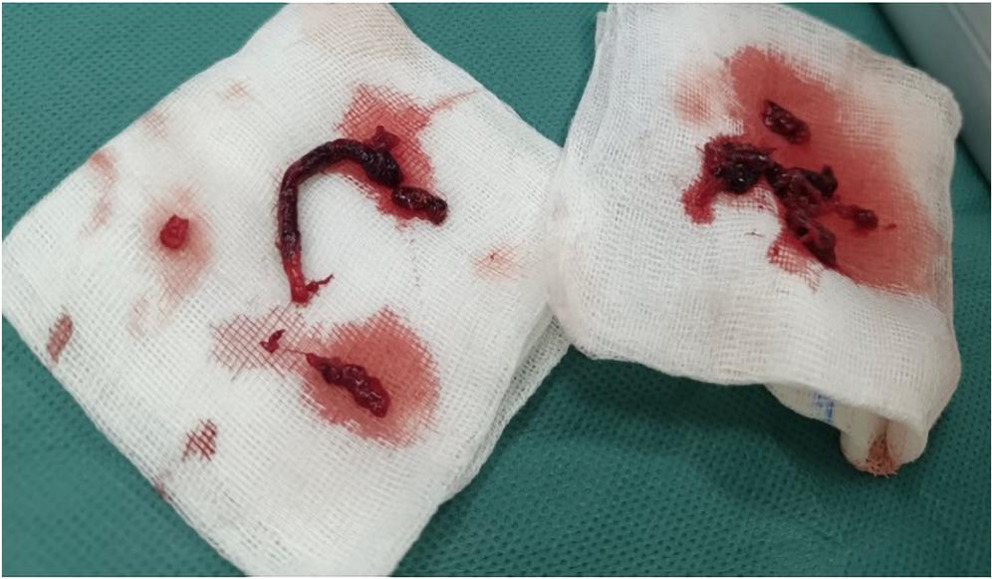
Image of thrombi removed.

The patient then received a bolus of 5000IU unfractionated heparin and was rapidly transferred to the operating room for Fogarty embolectomy with removal of endoluminal thrombi (*Figure 3*).

An arterial echo-Doppler of the lower limbs was performed postoperatively and confirmed the success of Fogartization, showing permeable arteries in both lower limbs, with normal flow and velocity.

**Figure 4:**
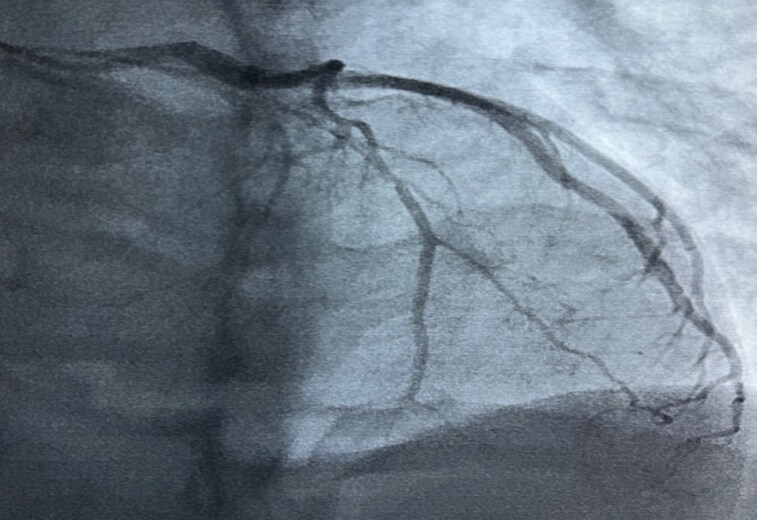
Left anterior oblique-cranial coronary angiography image showing thrombotic occlusion of the left anterior descending artery.

The patient underwent coronary artery angiography, which revealed thrombotic occlusion of the proximal left anterior descending artery (*Figure 4*).

**Figure 5:**
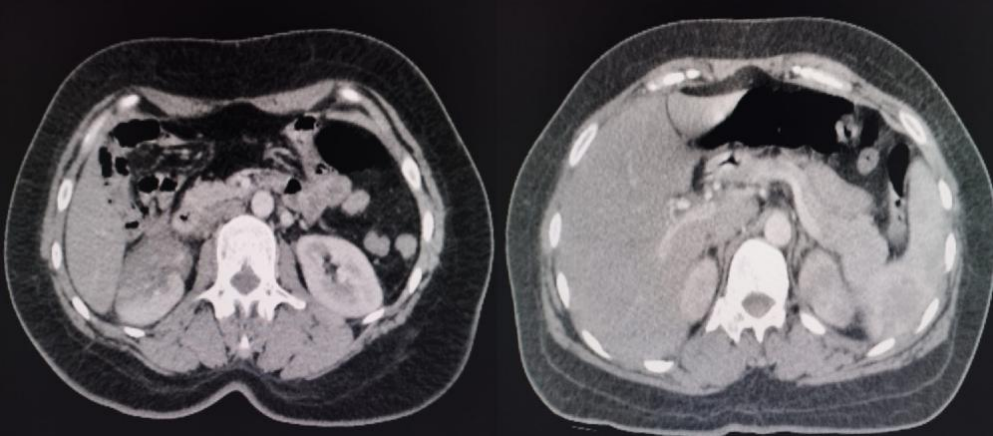
Computed tomography image showing right renal infarct (image in the left) and splenic infarct (image in the right).

A cerebro-thoraco-abdomino-pelvic computed tomography scan was performed as part of the search for subclinical systemic embolisms, finding a right renal infarct and a splenic infarct (*Figure 5*).

A work-up was requested to look for coagulopathy or thrombophilia. This work-up revealed positive anti-cardiolipin antibodies (aCL) (IgG and IgM). The rest of the immunological work-up and thrombophilia work-up were negative, including lupus anticoagulant (LA), anti-β2-glycoprotein I antibodies, with normal protein C, S, and antithrombin.

Anti-phospholipid syndrome was confirmed on the basis of all the clinical and biological arguments raised, and the patient was put on vitamin K antagonists with international normalized ratio (INR) monitoring. Clopidogrel was stopped and low dose aspirin maintained, given the high ischaemic risk. The rest of the treatment for heart failure with reduced ejection fraction was maintained, namely Bisoprolol, Ramipril, and Spironolactone, with the introduction of Dapagliflozin.

The evolution was marked by complete resolution of LV thrombi confirmed by transthoracic echocardiogram after 3 weeks of medical treatment. The patient is currently asymptomatic apart from a slight exertional dyspnoea.

## Discussion

Anti-phospholipid syndrome is a chronic inflammatory disease characterized by arterial and venous thrombosis, often associated with high titres of anti-phospholipid antibodies (APL).^4^ These antibodies are a diverse group of antibodies that share the common feature of being directed against anti-phospholipid-related proteins. This group includes LA, aCL, and anti-β2-glycoprotein I (anti-β2GPI).^5,6^

Thrombotic events are a major source of morbidity and mortality in patients with APS. Venous thromboembolism is the first manifestation of this pathology, but it should not be overlooked that a large proportion of patients suffer from arterial damage (acute coronary syndrome, stroke, etc.). Indeed, Vianna *et al.*^7^ reported episodes of deep vein thrombosis in 54% of cases while arterial occlusions occur in 44% of cases in patients with APS.

The incidence of myocardial infarction in patients with APS is estimated at 4%. In a prospective multicentre cohort of 1000 patients with APS, myocardial infarction was the revealing event in 2.8% of patients, and occurred during follow-up in 5.5%.^8^

In addition to APL antibodies, known cardiovascular risk factors may coexist in these patients, increasing the risk of thrombosis.^9^ In our patient’s case, this was not the case at all, as she had no cardiovascular risk factors.

When patients present with severe and dramatic APS with extensive diffuse embolization, as in our case, treatment with methylprednisone or more aggressive therapies such as Plasmapheresis, Immunoglobulin or Cyclophosphamide may be proposed.^10^

In the presence of intracardiac thrombus, as in our patient’s case, it was formerly recommended, years before, to adopt a more intensive anticoagulation regimen based on vitamin K antagonists, with INR targets of between 3 and 4,^11^ whereas a more recent study^12^ demonstrated the absence of benefit from this more aggressive anticoagulation and proved that the standard target, between 2 and 3, should be aimed in similar situations.

For the management of ST-segment elevation myocardial infarction, studies are few and far between, and the cases reported in the literature suggest that the results of percutaneous angioplasty and coronary artery bypass grafting are less good in patients with APS, given the frequency of stent thrombosis or bypass failure.^13^ However, primary percutaneous angioplasty and dual antiplatelet therapy remain the standard of care for patients with APS and ST-segment elevation myocardial infarction.^13^ Patients with APS and ST-segment elevation myocardial infarction should undergo percutaneous coronary intervention, usually combined with thrombus aspiration when the thrombotic load is high, and in certain situations, drug-eluting stent implantation. After stent implantation, triple antithrombotic therapy with dual short-term antiplatelet therapy and long-term anticoagulant therapy is recommended. Clinicians should include an aetiological investigation in the workup of their patients admitted for myocardial infarction, especially when the terrain is atypical, and particularly in young subjects and women.^14^

In terms of chronic management, more aggressive treatment of all atherosclerosis risk factors (hypertension, diabetes, hypercholesterolaemia, smoking…) in addition to folic acid, vitamin B and cholesterol-lowering drugs (preferably statins) are recommended in patients with APS.^10^

In primary prevention, treatment with low-dose aspirin has been shown to be effective not only in reducing the risk of a first thrombotic event in patients with APS but also in preventing recurrence in these patients.^15^ In case of recurrent thrombosis, increasing the intensity of warfarin anticoagulation to reach a higher target INR (target INR 2.5–3.5 or 3.0–4.0), moving from warfarin to therapeutic doses of unfractionated heparin or low-molecular-weight heparin, or combining warfarin with an antiplatelet agent, especially aspirin low dose, are some potential treatment options even when warfarin is in the target INR range.^15^

Based to a meta-analysis,^16^ the use of direct oral anticoagulants—mainly rivaroxaban—was associated with a 69% higher risk of thrombo-embolic events comparing to vitamin K antagonists (VKAs), especially in patients with high-risk APS profiles. On the other hand, there was no significant difference in the two groups in terms of risks of major bleeding or death. Therefore, VKAs should remain to the first-line treatment for patients with APS, especially those with a high-risk profile.

One hundred and ninety four patients with anti-phospholipid antibodies (aPL), including 112 with APS symptoms and 82 asymptomatic carriers of the disease, had their factor XI levels evaluated in a recent study.^17^ According to the findings, factor XI levels were noticeably higher in APS patients than in asymptomatic carriers. On the other hand, APS patients had fewer instances of low factor XI levels, indicating a possible defense against thrombosis. According to these findings, factor XI may be a contributing factor to the prothrombotic state brought on by aPL, and focusing on FXI may provide APS patients with an additional therapeutic option.

The characteristic presentation of APS is venous and/or arterial thrombosis, any organ system or vessel may be impacted by one or more events, concurrently or consecutively.^18^ In a meta-analysis^19^ totalling 16 441 patients, it was found that aPL, particularly LA and aCL, increased significantly the risk of venous thrombosis. As of arterial thrombosis, they were highly associated with APS, as LA had an odds ratio (OR) of 3.58, anti-cardiolipin had an OR of 2.65, anti-β2-glycoprotein I antibodies had an OR of 3.12, and anti-phosphatidylserine antibodies had an OR of 6.00.^19^ Based on all of these studies and evidence, APS may be a true risk factor for venous and/or arterial thrombosis.

## Conclusion

Anti-phospholipid syndrome is an autoimmune disorder whose complications can be life-threatening and/or functionally disabling. Arterial thrombosis can cause dramatic complications.

## Data Availability

No new data were generated or analysed in support of this article.

## Lead author biography

Professor Meryem Haboub, Assistant Professor of Cardiology in the Faculty of Medicine, Hassan II University Casablanca, Morocco.
